# Oral Sodium Hyaluronate Reshapes Gut Microbiota Composition and Suppresses the LPS-TLR4/NF-κB Pathway to Exert Neuroprotection in MPTP-Induced Parkinson’s Disease

**DOI:** 10.3390/ijms27156573

**Published:** 2026-07-23

**Authors:** Yishu Wang, Zhi Cao, Chao Zhang, Yong Ying, Gaofei Zhu, Jie Wang, Xinyu Hou, Daizhou Zhang, Zhiyong Zheng, Huarong Shao, Fei Liu, Xiaodong Ma

**Affiliations:** 1 Key Laboratory of Brain, Cognition and Education Sciences, Ministry of Education, Institute for Brain Research and Rehabilitation, South China Normal University, Guangzhou 510631, China; 2021010276@m.scnu.edu.cn (Y.W.); caozhi420@whu.edu.cn (Z.C.); 2 Shandong Academy of Pharmaceutical Sciences, Engineering Research Center for Sugar and Sugar Complex, National-Local Joint Engineering Laboratory of Polysaccharide Drugs, Key Laboratory of Carbohydrate and Glycoconjugate Drugs, Jinan 250101, China; 17860504439@163.com (C.Z.); yingyong@sdaps.cn (Y.Y.); gor17860504646@163.com (G.Z.); wangjie91013@163.com (J.W.); 13258676515@163.com (X.H.); zhangdaizhou@sdaps.cn (D.Z.); zhengzhiyong@sdaps.cn (Z.Z.); shaohuarong@sdaps.cn (H.S.); 3 The Second Clinical Medical College, Jinan University, Shenzhen 518020, China; 4 College of Pharmacy, Shandong University of Traditional Chinese Medicine, Jinan 250355, China

**Keywords:** sodium hyaluronate, Parkinson’s disease, neuroprotection, microbiota–gut–brain axis, neuroinflammation

## Abstract

Parkinson’s disease (PD), a debilitating neurodegenerative disorder, is primarily characterized by motor impairments and concurrent gastrointestinal disturbances. Increasing evidence has highlighted the critical role of the microbiota–gut–brain axis (MGBA) in the pathogenesis of PD. This study investigated the neuroprotective potential of sodium hyaluronate (SH) in a mouse model of PD and its underlying mechanisms via the MGBA. In the oral pre-treatment study, three doses (7.5, 15, and 30 mg/kg/day) were evaluated. The results showed that the high dose (SH-H, 30 mg/kg/day) significantly ameliorated motor disorders and gastrointestinal functional disorders. Therefore, SH-H was selected for subsequent mechanistic investigations. Mechanistically, SH-H restored gut microbiota homeostasis, increased fecal short-chain fatty acid (SCFA) levels, and improved the integrity of the intestinal and blood–brain barrier (BBB). Thus, SH reduced the transfer of lipopolysaccharide (LPS) from the intestine to serum and the substantia nigra (SN), suppressing activation of the LPS-TLR4/MyD88/NF-κB signaling pathway. These effects alleviated neuroinflammation, protected dopaminergic neurons, and reduced the aggregation of α-synuclein (α-syn). In summary, SH attenuated PD-related pathological changes by restoring gut microbiota homeostasis and modulating the MGBA, suggesting that SH may represent a potential therapeutic strategy for PD.

## 1. Introduction

Parkinson’s disease (PD) is a progressive neurodegenerative disorder increasingly recognized for its strong association with gastrointestinal dysfunction [1,2,3]. A common hypothesis proposes that PD may begin in the gut. In this scenario, microbial imbalance compromises the intestinal barrier, allowing pro-inflammatory molecules like lipopolysaccharide (LPS) to enter the bloodstream [4,5,6]. This process can then set off a neuroinflammatory cascade, which ultimately contributes to the loss of dopaminergic neurons in the substantia nigra (SN) [7]. Despite the central role of this axis in PD pathogenesis, targeted therapeutic strategies remain underdeveloped. Thus, interventions aimed at restoring gut barrier integrity and reducing gut-driven neuroinflammation represent a promising therapeutic direction for PD.

Sodium hyaluronate (SH) is a glycosaminoglycan (GAG) found in the extracellular matrix of cells [8]. It is widely recognized for its roles in maintaining tissue homeostasis, exerting anti-inflammatory and antioxidant effects, and promoting tissue repair [9,10]. Increasing evidence highlights its critical role in gastrointestinal health, particularly in preserving gut barrier integrity and modulating microbiota composition [11,12]. These properties make SH an ideal candidate drug for the intervention of PD. SH has been proven to improve intestinal permeability in colitis models and reduce pro-inflammatory signals in liver injury models, indicating its ability to provide barrier protection and immune regulation [13,14]. Therefore, SH may intercept the key drivers of PD pathology on the microbiota–gut–brain axis (MGBA) by enhancing the intestinal barrier, reducing systemic inflammatory responses, and promoting microbial balance.

It is worth noting that SH is an endogenous substance [15], and it has been safely orally used as a food ingredient in multiple regions, which significantly enhances its feasibility for application [16]. Therefore, oral administration of SH becomes a reasonable and promising strategy to target the intestinal-mediated inflammation and barrier dysfunction in PD, opening up a new avenue for the treatment of PD and worthy of further research.

We propose that oral administration of SH may reduce the production of harmful substances by maintaining the stability of the intestinal microbiota and reducing the entry of inflammatory factors into the bloodstream by repairing the intestinal barrier, correcting intestinal dysfunction, and inhibiting intestinal inflammatory responses. This, in turn, can prevent the activation of microglia and the infiltration of inflammatory factors into the blood–brain barrier (BBB), thereby preventing the occurrence of neurological inflammation. Thus, it can play a preventive role in the motor and non-motor symptoms of PD in mice. To address this issue and gain a comprehensive understanding, we used a PD mouse model induced by 1-methyl-4-phenyl-1,2,3,6-tetrahydropyridine (MPTP) to evaluate the preventive effect of SH on PD and to deeply explore its underlying mechanism.

## 2. Results

### 2.1. Oral SH Administration Alleviated Motor Symptoms and Gastrointestinal Dysfunction in the MPTP-Induced PD Mouse Model

**Figure 1:**
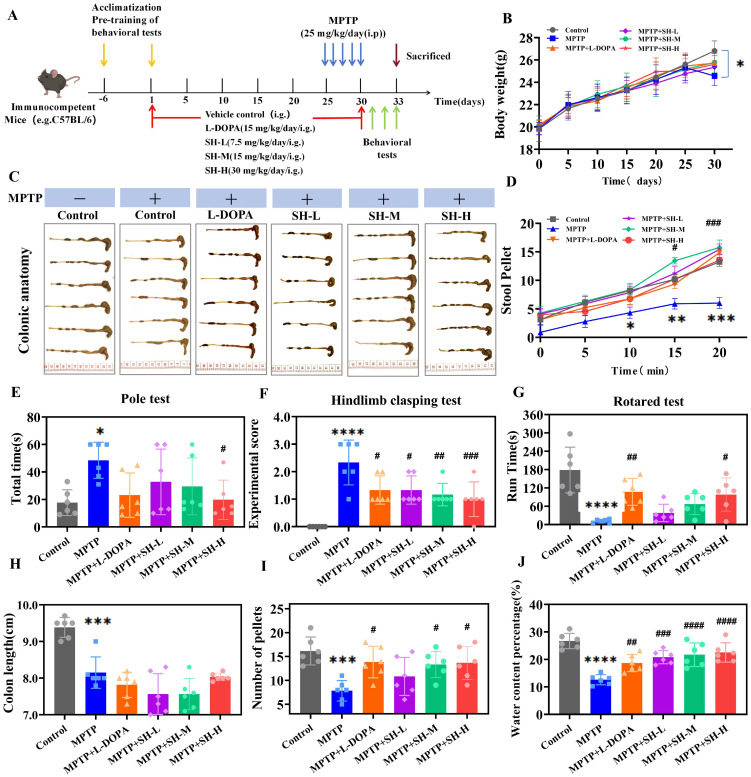
Oral SH alleviates motor symptoms and gastrointestinal dysfunction in MPTP-induced PD mouse models. (**A**) The procedure of animal administrations with oral SH treatment. (**B**) The body weights of mice. (**C**) Pictures of mice colon. (**D**) Time course of fecal output over 20 min. (**E**) Climbing time from top to bottom of a pole. (**F**) Scores of hindlimb clasping test. (**G**) Latency time on the rotating rod. (**H**) Colonic tissue length. (**I**) Total fecal output. (**J**) Stool water percentage. (For (**B**–**J**), n = 6 for each group. Data are presented as mean ± SD. * *p* < 0.05, ** *p* < 0.01, *** *p* < 0.001, **** *p* < 0.0001 versus the control group; # *p* < 0.05, ## *p* < 0.01, ### *p* < 0.001, #### *p* < 0.0001 versus the MPTP group.).

To accurately investigate the effects of oral SH on PD symptoms, including behavioral performance and intestinal function, we designed a comprehensive study. The mice were randomly divided into four groups and given different concentrations of SH (SH-L, SH-M, SH-H) and L-DOPA by intragastric administration for 4 weeks. During the last 5 days of the regimen, PD mouse models were successfully constructed by intraperitoneal injection of MPTP (Figure 1A).

As expected, mice in the MPTP-treated group lost significant weight after modeling, while those treated with SH and L-DOPA tended to have normal weight (Figure 1B, one-way ANOVA, F (5, 54) = 14.85, *p* < 0.0001). From day 30, three key behavioral tests were performed to assess motor function in each group of mice: a pole climbing test (Figure 1E, one-way ANOVA, F (5, 30) = 2.672, *p* = 0.0411) to assess motor balance, a hindlimb grip reflex test (Figure 1F, one-way ANOVA, F (5, 30) = 11.42, *p* < 0.0001) to monitor disease progression, and a rotating-rod test (Figure 1G, one-way ANOVA, F (5, 30) = 10.00, *p* < 0.0001) to measure motor coordination. Compared with the control group, MPTP-induced mice showed significant dyskinesia, extended pole climbing time, significantly increased rear limb grip reflex, and shortened retention time on the rotating rod. Fortunately, after taking SH orally, the motor disorder was relieved.

Gastrointestinal function was assessed after oral SH treatment. MPTP mice displayed colonic narrowing, fecal deformation (Figure 1C), and significant colonic shortening versus controls (Figure 1H; F (5, 30) = 18.87, *p* < 0.0001). Quantitative analyses revealed that MPTP mice had significantly fewer fecal pellets (Figure 1I; F(5, 30) = 5.079, *p* = 0.0017), lower fecal water content (Figure 1J; F (5, 30) = 13.78, *p* < 0.0001), and reduced defecation frequency (Figure 1D; F (4, 20) = 65.21, *p* < 0.0001) than controls. Both L-DOPA and SH partially improved these macroscopic abnormalities.

In summary, these results clearly demonstrate that MPTP induces weight loss, movement disorders, and gastrointestinal dysfunction in a mouse model of PD. Results from the initial dose-screening phase indicated that oral administration of SH significantly alleviated PD-related symptoms in a dose-dependent manner, with the high dose (SH-H, 30 mg/kg/day) exerting the most pronounced therapeutic effects. These findings highlight the potential of SH, particularly at the 30 mg/kg/day dose, as a candidate intervention for PD. Therefore, we chose SH-H (30 mg/kg) as the concentration for follow-up experiments to conduct relevant studies on the action mechanism.

### 2.2. Oral SH Administration Altered the Gut Microbiota Composition and SCFAs Production in PD-like Mice

**Figure 2:**
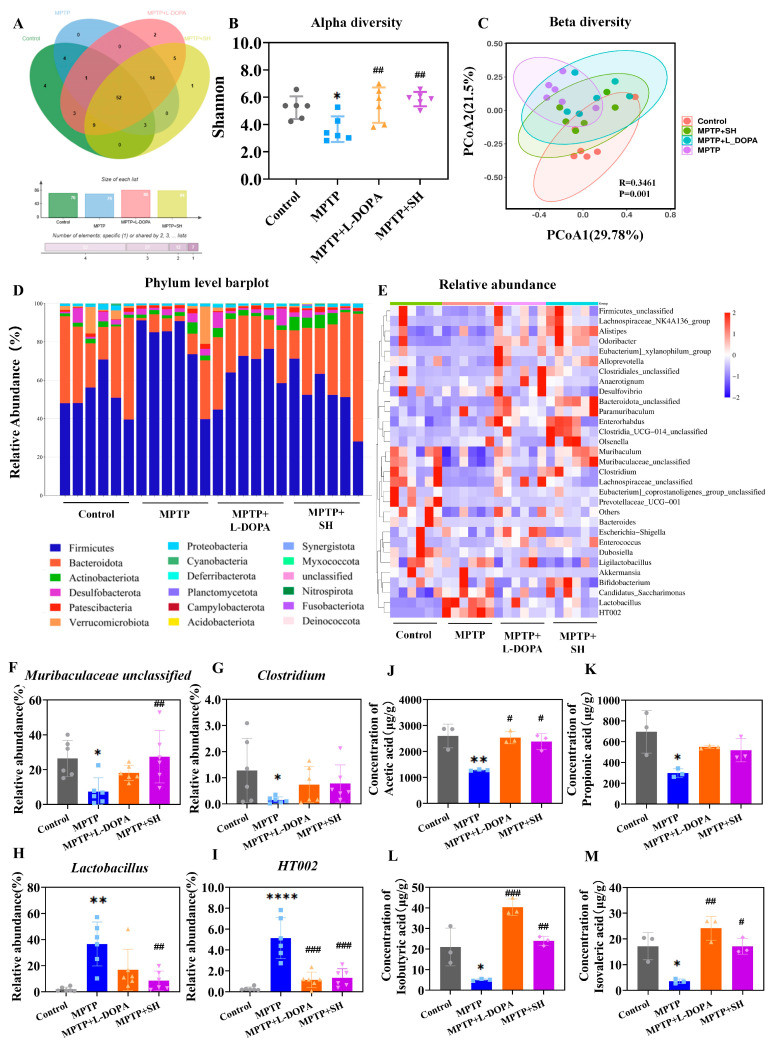
Oral SH administration rectified the dysbiosis of the fecal microbiota in MPTP-induced PD mouse models. (**A**) The Venn diagram among the different groups. (**B**) Analysis of alpha diversity of the gut microbiota by Observed-OTU analysis. (**C**) PCoA plots of beta diversity based on the Bray–Curtis distances analysis in different groups. (**D**) Relative abundances of gut microbiota at the phylum level in the 4 groups. (**E**) Heatmap analysis of relative abundances of gut microbiota at the genus level in different groups. (**F**–**I**) Relative abundances of 4 significantly altered bacterial genera: *Muribaculaceae unclassified* (**F**), *Clostridium* (**G**), *Lactobacillus* (**H**), and *HT002* (**I**). (**J**–**M**) The contents of acetic acid, propionic acid, isobutyric acid, and isovaleric acid in feces. (In this figure, *n* = 6 for each group. Data are presented as mean ± SD. * *p* < 0.05, ** *p* < 0.01, **** *p* < 0.0001 versus the control group; # *p* < 0.05, ## *p* < 0.01, ### *p* < 0.001 versus the MPTP group.).

To delineate the impact of SH intervention on the gut microbiota of PD mice, we conducted 16S rDNA sequencing on fecal samples from the mice (Figure 2, Table S1). We visualize the overlap and uniqueness between different datasets through a Venn diagram (Figure 2A). The observed species index primarily estimates the number of species within a community, reflecting the α-diversity of the microbiota. Compared to the control group, the MPTP group exhibited a significant reduction in this index, while a trend of increase was observed in the L-DOPA and SH groups, suggesting alterations in the gut microbiota of PD mice following SH intervention (Figure 2B; one-way ANOVA, F (3, 20) = 6.272, *p* = 0.0036). Moreover, to assess β-diversity, we performed Principal Coordinates Analysis (PCoA) based on Bray-Curtis distances. The result indicated differences between the control and MPTP groups. Although some points overlapped with the MPTP group, the samples from the L-DOPA and SH treatment groups tended to cluster with the control group. Statistical significance of β-diversity differences among groups was evaluated using ANOSIM (analysis of similarities) with 999 permutations. The overall ANOSIM result revealed significant differences among the four groups (R = 0.3461, *p* = 0.001). The same clustering pattern was also confirmed using PCA based on weighted UniFrac distances (Figure S2), confirming that our conclusions are robust regardless of the analytical method employed.

At the phylum level, the bacterial community in the control group was predominantly composed of *Firmicutes* (52.25%), *Bacteroidota* (35.85%), *Verrucomicrobiota* (3.76%), and *Desulfobacterota* (3.07%), with minor contributions from *Actinobacteriota* (1.92%) and *Proteobacteria* (1.69%). As depicted, the gut microbiota of MPTP-induced PD mice showed an increase in *Firmicutes* to 77.61% and a decrease in *Bacteroidota* to 10.2%, which was reversed following L-DOPA and SH treatments (Figure 2D).

Analysis of the relative proportions of the top 30 bacterial genera revealed that the most abundant genera included *Ligilactobacillus, Muribaculaceae_unclassified, Akkermansia, Dubosiella, Desulfovibrio, Lachnospiraceae_unclassified, Muribaculum, Bacteroides, Lachnospiraceae_NK4A136_group, Clostridiales_unclassified,* and *Lactobacillus.* Detailed heatmap analysis at the genus level indicated significant changes in the gut microbiota during the neurodegenerative process in PD-like mice (Figure 2E). In particular, compared to the control group, the MPTP group exhibited lower relative abundances of *Muribaculaceae_unclassified* (Figure 2F, one-way ANOVA, F (3, 20) = 4.950, *p* = 0.0099) and *Clostridium* (Figure 2G, one-way ANOVA, F (3, 20) = 2.032, *p* = 0.1417) and higher relative abundances of *Lactobacillus* (Figure 2H, one-way ANOVA, F (3, 20) = 9.178, *p* = 0.0005) and *HT002* (Figure 2I, one-way ANOVA, F (3, 20) = 21.66, *p* < 0.0001). L-DOPA and SH interventions partially restored the abundance levels of these genera.

SCFAs, particularly acetic acid, propionic acid, and butyric acid, are the principal end products of bacterial fiber fermentation in the gut [17]. They exert diverse effects on the host, typically regulating epithelial barrier function and mucosal and systemic immunity [18], and have been demonstrated to provide direct or indirect neuroprotective benefits to peripheral and central functions [19]. SCFAs are considered key mediators in the MGBA [20]. A study comparing fecal SCFAs levels between PD patients and controls found that reduced SCFAs concentrations in PD were associated with alterations in the microbiota [21]. LC-MS/MS analysis revealed an overall decrease in fecal SCFAs in PD mice, with significant reductions in acetic acid (Figure 2J, one-way ANOVA, F (3, 8) = 12.61, *p* = 0.0021), propionic acid (Figure 2K, one-way ANOVA, F (3, 8) = 5.676, *p* = 0.0222), isobutyric acid (Figure 2L, one-way ANOVA, F (3, 8) = 23.67, *p* = 0.0002), and isovaleric acid (Figure 2M, one-way ANOVA, F (3, 8) = 15.06, *p* = 0.0012) compared to the control group. As anticipated, the L-DOPA and SH groups reversed these trends.

### 2.3. Oral SH Administration Mitigated BBB and Intestinal Barrier Dysfunction in the MPTP-Induced PD Mouse Model

The integrity of the BBB and the intestinal barrier is fundamental to neural health and gastrointestinal function, both of which are compromised in PD [22]. The disruption of these barriers is a critical aspect of PD pathology, contributing to neurodegeneration and gastrointestinal dysregulation. To address this, we investigated the impact of SH on barrier function in a mouse model of PD induced by MPTP.

**Figure 3:**
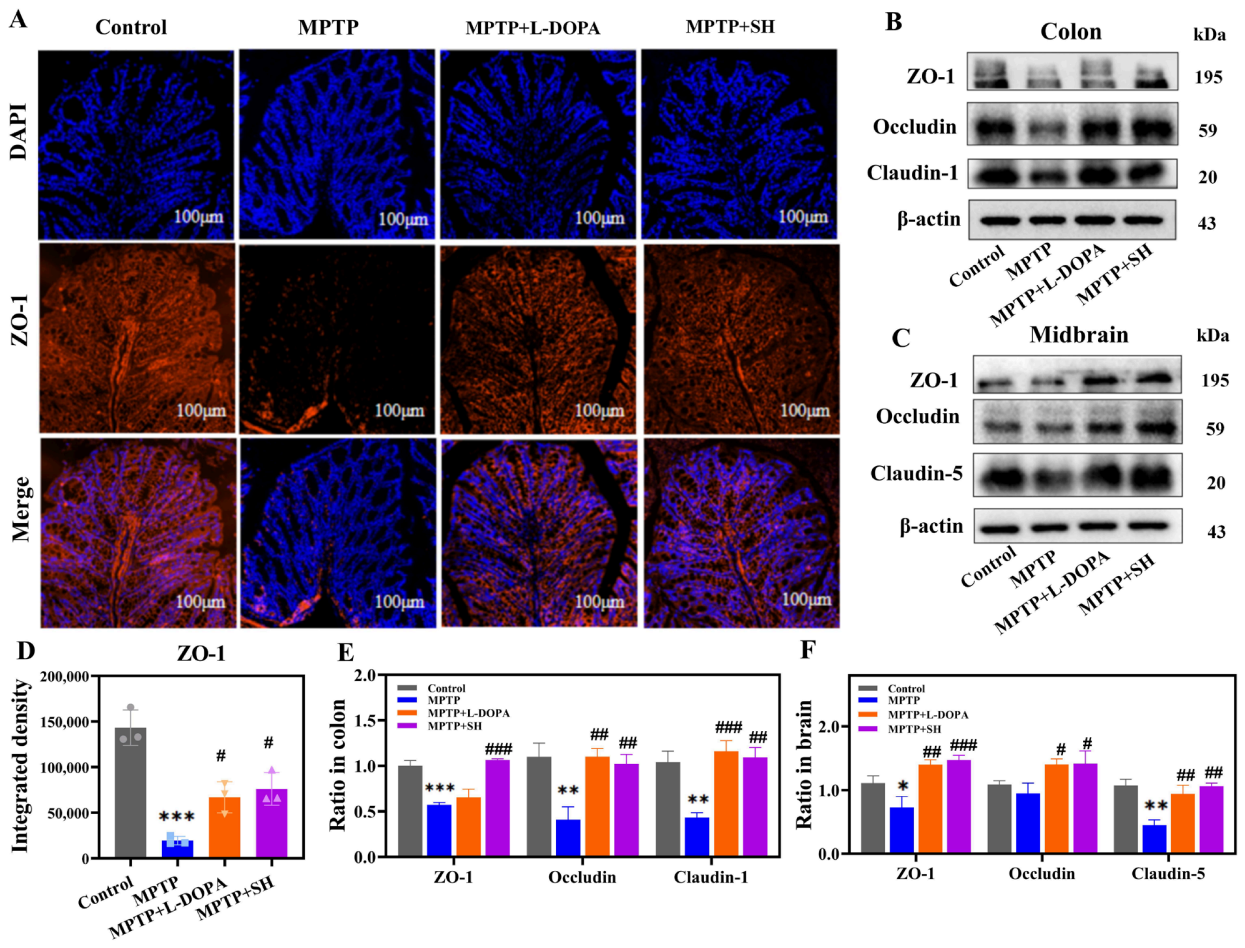
Oral SH restored the BBB and intestinal barrier impairment in the MPTP-induced PD mouse models. (**A**) Representative captures of immunofluorescence in the colon of ZO-1. (**B**) Representative WB bands of ZO-1, Occludin, and Claudin-1 in the colon. (**C**) Representative WB bands of ZO-1, Occludin, and Claudin-5 in the midbrain containing the SN. (**D**) ZO-1 integrity score in the colon. (**E**) The density analysis of ZO-1, Occludin, and Claudin-1 WB in the colon. (**F**) The density analysis of ZO-1, Occludin, and Claudin-5 WBs in the midbrain containing the SN. (In this figure, *n* = 3 for each group. Data are presented as mean ± SD. * *p* < 0.05, ** *p* < 0.01, *** *p* < 0.001 versus the control group; # *p* < 0.05, ## *p* < 0.01, ### *p* < 0.001 versus the MPTP group.).

Our immunofluorescence staining was employed to assess the expression and distribution of ZO-1 in the colonic epithelium. MPTP treatment led to a disrupted and reduced expression of ZO-1, which was significantly improved following SH administration (Figure 3A,D; one-way ANOVA, F (3, 8) = 30.66, *p* < 0.0001). To further validate this finding, Western blot (WB) analyses revealed a significant decrease in the expression of tight junction proteins, including ZO-1 (one-way ANOVA, F (2, 6) = 2.314, *p* = 0.0001), Occludin (one-way ANOVA, F (2, 6) = 0.3199, *p* = 0.002), and Claudin-1 (one-way ANOVA, F (2, 6) = 0.1467, *p* = 0.0018) in the colonic tissues of MPTP-challenged mice compared to controls. This reduction indicates a compromised intestinal barrier. However, treatment with oral SH resulted in a marked upregulation of these proteins, suggesting a restoration of barrier integrity (Figure 3B,E). Similarly, in the midbrain SN, a region highly susceptible to PD-related pathology, we observed a significant decrease in the expression of tight junction proteins such as ZO-1 (one-way ANOVA, F (2, 6) = 0.3421, *p* = 0.02), Occludin (one-way ANOVA, F (2, 6) = 0.2025, *p* = 0.5822), and Claudin-5 (one-way ANOVA, F (2, 6) = 0.04741, *p* = 0.0011) in MPTP-exposed mice. SH treatment effectively reversed this trend, with a marked upregulation in the expression of ZO-1, Occludin, and Claudin-5 (Figure 3C,F).

Collectively, these data demonstrate that oral SH administration can ameliorate the BBB and intestinal barrier dysfunction induced by MPTP in a PD mouse model. This therapeutic intervention may represent a novel strategy for targeting the multifaceted pathophysiology of PD, warranting further investigation into its action mechanisms and potential clinical application.

### 2.4. Oral SH Administration Mitigated PD-Associated Histological Features and Inflammatory Responses in the MPTP-Induced Mouse Model

**Figure 4:**
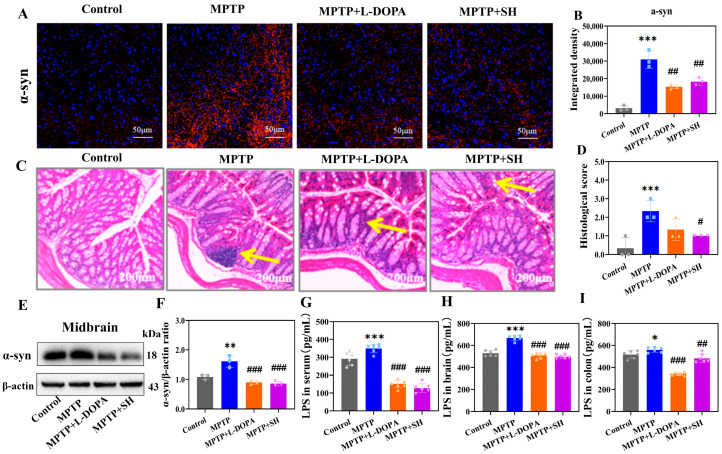
Oral SH administration mitigated PD-associated histological features and inflammatory responses in the MPTP-induced mouse model. (**A**) Representative colonic immunofluorescence images of α-syn (DAPI, blue; α-syn, red). (**B**) α-syn integrity score in brain tissues. (**C**) H&E-stained colon sections, with yellow arrows indicating inflammatory infiltration. (**D**) Histological scores derived from colonic H&E staining. (**E**) WB detection of α-syn in the SN. (**F**) Densitometric analysis of α-syn protein bands. (**G**–**I**) LPS endotoxin levels in serum (**G**), midbrain containing the SN (**H**), and colon (**I**). Sample sizes: n = 3 per group for (**A**–**F**), n = 6 per group for (**G**–**I**). (Data are presented as mean ± SD. * *p* < 0.05, ** *p* < 0.01, *** *p* < 0.001 versus the control group; # *p* < 0.05, ## *p* < 0.01, ### *p* < 0.001 versus the MPTP group.).

To further explore how oral SH alleviates PD symptoms, we conducted detailed morphological observations of the colon and midbrain, along with the detection of corresponding inflammatory factors and specific biomarkers. The aggregation of α-syn is widely recognized as one of the important histopathological markers of PD [23,24]. In the present study, immunofluorescence staining confirmed that the expression of α-syn was significantly increased in the MPTP group compared to the control group, and this reduction was reversed by SH treatment (Figure 4A,B; one-way ANOVA, F (3, 8) = 47.89, *p* < 0.0001). Consistently, midbrain protein blotting showed the same results (Figure 4E,F; one-way ANOVA, F (3, 8) = 25.10, *p* = 0.0002). Immunoinfiltration and epithelial damage in the colon were assessed by H&E staining (Figure 4C,D; one-way ANOVA, F (3, 8) = 8.333, *p* = 0.0076). As depicted, the MPTP group exhibited marked inflammatory infiltration, with the H&E histological scores significantly higher than those of the control group, whereas that was markedly reduced by oral SH administration. Therefore, we assessed the severity of the inflammatory response with LPS treatment. ELISA analysis revealed that serum LPS (Figure 4G, one-way ANOVA, F (3, 20) = 91.42, *p* < 0.0001), midbrain LPS (Figure 4H, one-way ANOVA, F (3, 20) = 70.02, *p* < 0.0001), and colon LPS (Figure 4I, one-way ANOVA, F (3, 20) = 60.67, *p* < 0.0001) were all significantly elevated in the MPTP group compared to the control group. Following oral SH administration, the levels of LPS decreased significantly. Moreover, compared to the control group, the MPTP group exhibited significantly higher levels of pro-inflammatory cytokines (TNF-α and IL-6), which were effectively reduced by SH administration (Figure S1, TNF-α in serum, one-way ANOVA, F (3, 20) = 15.05, *p* < 0.0001; TNF-α in midbrain, one-way ANOVA, F (3, 20) = 224.8, *p* < 0.0001; TNF-α in colon, one-way ANOVA, F (3, 20) = 56.91, *p* < 0.0001; IL-6 in serum, one-way ANOVA, F (3, 20) = 59.06, *p* < 0.0001; IL-6 in midbrain, one-way ANOVA, F (3, 20) = 103.8, *p* < 0.0001; IL-6 in colon, one-way ANOVA, F (3, 20) = 46.87, *p* < 0.0001).

These results effectively confirm that oral administration of SH can alleviate systemic inflammation by reducing the levels of inflammatory factors and can also minimize the entry of harmful substances into the bloodstream and reduce the impact of inflammation on the brain.

**Figure 5:**
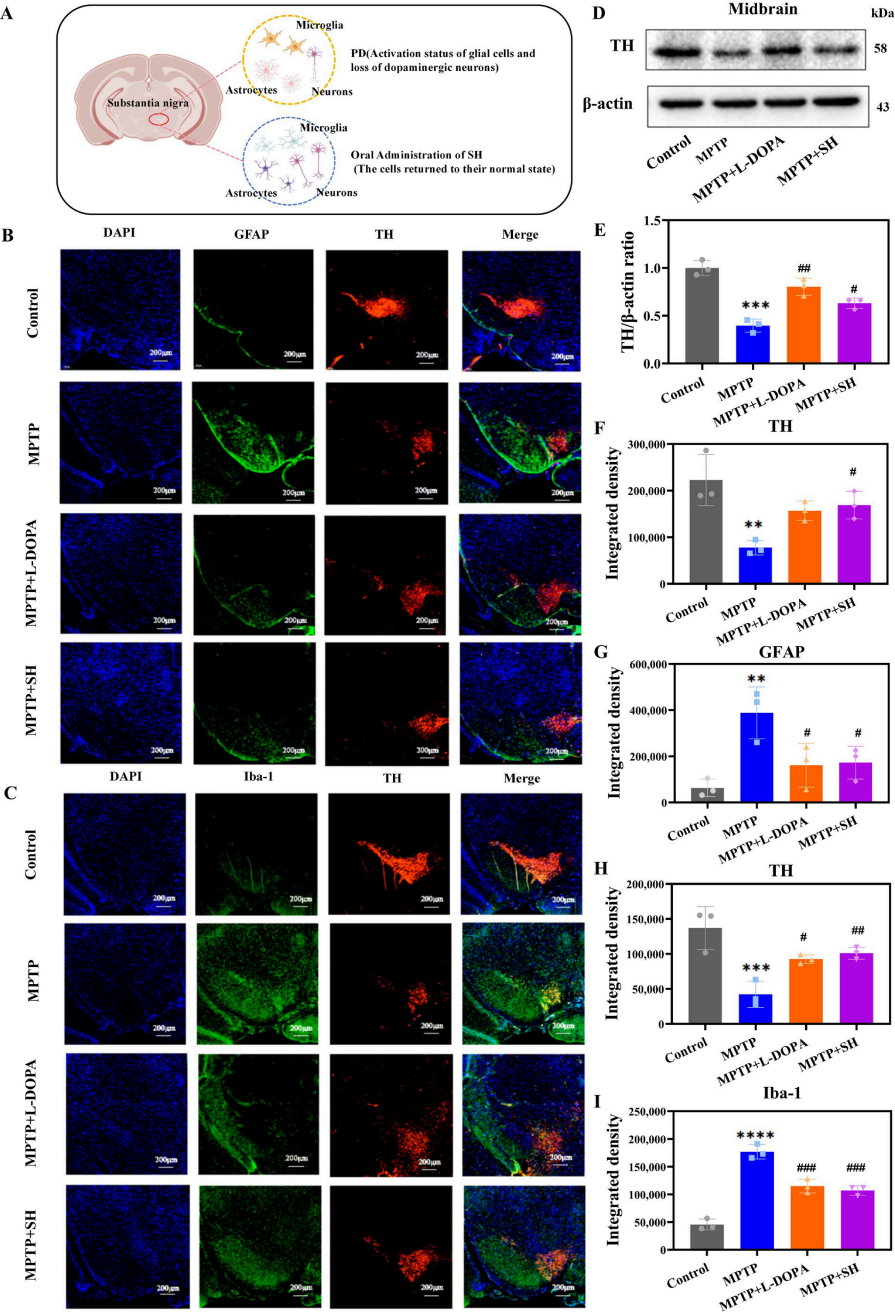
Oral SH mitigated neuronal damage and PD-related neuroinflammatory responses in the SN and colon in MPTP mouse models. (**A**) Schematic representation of the neuronal state in the PD model and after oral SH administration. (**B**) Representative captures of immunofluorescence in the SN of nuclei (DAPI, blue), total dopaminergic neurons (TH, red), and astrocytes (GFAP, green). (**C**) Representative captures of immunofluorescence in the SN of nuclei (DAPI, blue), total dopaminergic neurons (TH, red), and microglial cells (Iba-1, green). (**D**) WB for TH expression in SN tissue. (**E**) The density analysis of TH. (**F**) Integrated density of TH in the SN. (**G**) Integrated density of GFAP (activated astrocytes) in the SN. (**H**) Integrated density of TH in the SN. (**I**) Integrated density of Iba-1 (activated microglial cells) in the SN. (In this figure, *n* = 3 for each group. Data are presented as mean ± SD. ** *p* < 0.01, *** *p* < 0.001, **** *p* < 0.0001 versus the control group; # *p* < 0.05, ## *p* < 0.01, ### *p* < 0.001 versus the MPTP group.).

When brain-resident innate immune cells (microglia and astrocytes) are activated, they release reactive oxygen species and pro-inflammatory factors that recruit peripheral immune cells across the compromised BBB into the brain to clear pathogens [25]. However, if this process becomes dysregulated, it can trigger chronic neuroinflammation, establishing a vicious cycle that leads to neuronal damage and fuels the onset and progression of neurodegenerative disorders such as PD [26]. The results of this study indicated that oral administration of SH can effectively inhibit the activation of microglia and astrocytes and reduce the damage to neuronal cells (Figure 5A).

In this study, we used GFAP as a marker for astrocytes and Iba-1 for microglia to perform immunofluorescent staining in the SN region to detect the activation of glial cells (Figure 5B,C). GFAP (Figure 5G, one-way ANOVA, F (3, 8) = 8.039, *p* = 0.0085) and Iba-1 (Figure 5I, one-way ANOVA, F (3, 8) = 70.00, *p* < 0.0001) cells were both significantly increased in the SN region of MPTP group mice. However, SH administration significantly attenuated the activation of GFAP cells and Iba-1 cells. Taken together, these results indicate that oral SH significantly mitigates PD-associated histological features and inflammation in the MPTP-induced PD mouse model.

Another key PD histological hallmark in the SN was a significant reduction in TH expression in MPTP-treated mice, as shown by WB of midbrain tissues (Figure 5D,E; one-way ANOVA, F (3, 8) = 35.82, *p* < 0.0001). Conversely, SH treatment markedly increased TH aggregation (Figure 5B,C). Moreover, TH-positive dopaminergic neurons in the SN dropped to less than half of control levels in MPTP-induced PD mice, and this loss was significantly reversed by SH treatment (Figure 5F, one-way ANOVA, F (3, 8) = 9.447, *p* = 0.0052) (Figure 5H, one-way ANOVA, F (3, 8) = 13.08, *p* = 0.0019).

### 2.5. Oral SH Administration Inhibited the LPS-TLR4/MyD88/NF-κB Signaling Pathway in the SN and Colon of the MPTP-Induced Mouse Model

We hypothesized that compromised barriers led to the leakage of pathogenic LPS, which might activate the immune system, exacerbate the inflammatory response and potentially progress to systemic inflammatory response syndrome [27].

**Figure 6:**
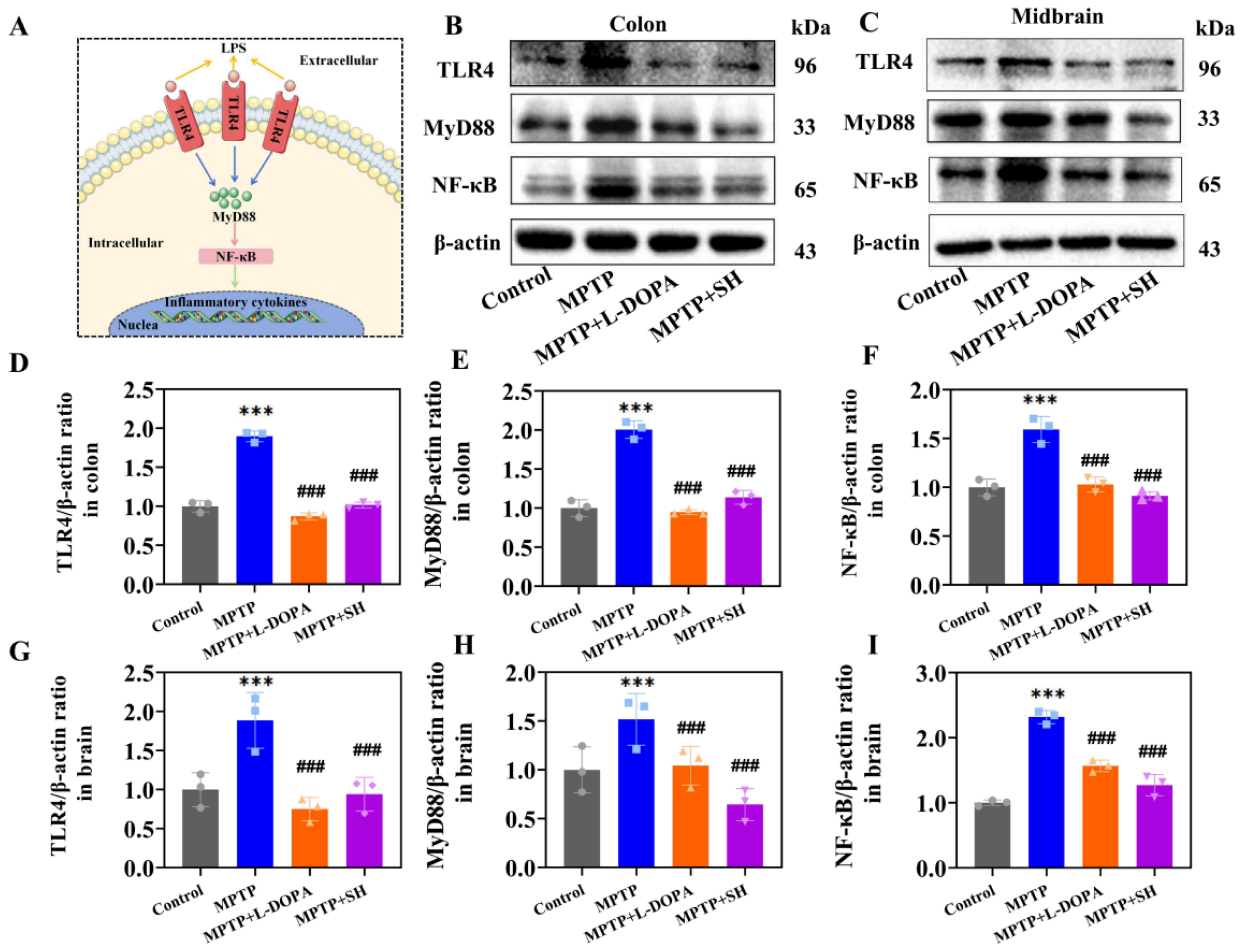
Oral SH inhibited the TLR4/MyD88/NF-κB signaling pathways both in the SN and the colon of MPTP-induced mice. (**A**) Schematic representation of the signaling pathway. (**B**) Representative WB bands of TLR4, MyD88, and NF-κB in the colon. (**C**) Representative WB bands of TLR4, MyD88, and NF-κB in the midbrain containing the SN. (**D**–**F**) The density analysis results of TLR4, MyD88, and NF-κB in the colon. (**G**–**I**) The density analysis results of TLR4, MyD88, and NF-κB in the midbrain containing the SN. (*n* = 3 for each group. Data are presented as mean ± SD. *** *p* < 0.001 versus the control group; ### *p* < 0.001 versus the MPTP group.).

TLR4 and its downstream signaling pathways are prominently advantageous in recognizing and responding to increased LPS [28]. Modulating LPS biosynthesis and LPS-TLR4 interactions can ameliorate gut dysbiosis and PD [29]. Given our finding of significantly elevated LPS levels in PD mice, we hypothesized that TLR4 could recognize these increased LPS molecules and activate a series of immune and inflammatory responses through its downstream signaling pathways (Figure 6A). WB confirmed TLR4 pathway activation in the colon (Figure 6B) and SN (Figure 6C), with consistent results across both tissues. MPTP group showed significant upregulation of TLR4, MyD88, and NF-κB versus controls (Figure 6D–F) (TLR4, one-way ANOVA, F (3, 8) = 198.2, *p* < 0.0001; MyD88, one-way ANOVA, F (3, 8) = 89.46, *p* < 0.0001; NF-κB, one-way ANOVA, F (3, 8) = 35.45, *p* < 0.0001), while SH treatment reversed this effect in the colon by reducing their expression.

Meanwhile, the results revealed significant upregulation of TLR4 (Figure 6G, one-way ANOVA, F (3, 8) = 12.63, *p* = 0.0021), MyD88 (Figure 6H, one-way ANOVA, F (3, 8) = 8.005, *p* = 0.0086), and NF-κB (Figure 6I, one-way ANOVA, F (3, 8) = 83.50, *p* < 0.0001) expression in the MPTP group in the midbrain containing the SN. Conversely, the SH group showed significant reduction in the expression of TLR4, MyD88, and NF-κB.

Taken together, these results demonstrate that oral SH treatment effectively blocks the LPS-stimulated TLR4/MyD88/NF-κB pathway in both colonic and nigral tissues of MPTP-intoxicated mice.

### 2.6. Oral SH Administration Inhibited the Production of Pro-Inflammatory Proteins in the SN and Colon of MPTP-Induced Mice

**Figure 7:**
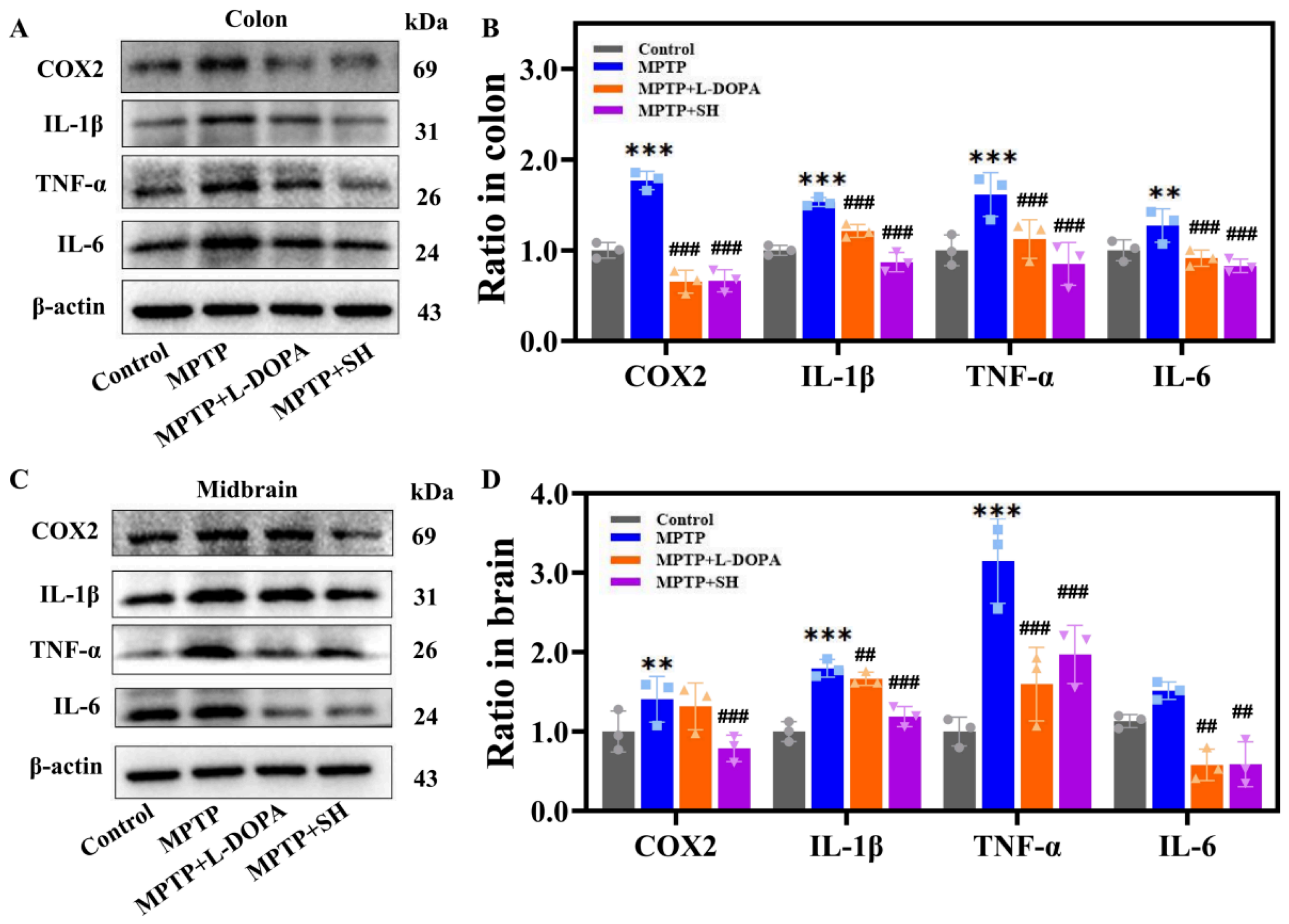
Oral SH suppressed the generation of pro-inflammatory molecules both in the SN and the colon of MPTP-induced mice. (**A**) Representative WB bands of TNF-α, IL-1β, IL-6, and COX-2 in the colon. (**B**) The density analysis results of TNF-α, IL-1β, IL-6, and COX-2 in the colon. (**C**) Representative WB bands of TNF-α, IL-1β, IL-6, and COX-2 in the midbrain containing the SN. (**D**) The density analysis results of TNF-α, IL-1β, IL-6, and COX-2 in the midbrain containing the SN. (*n* = 3 for each group. Data are presented as mean ± SD. ** *p* < 0.01, *** *p* < 0.001 versus the control group; ## *p* < 0.01, ### *p* < 0.001 versus the MPTP group.).

As a downstream effector of the TLR4/MyD88 axis, NF-κB triggers pro-inflammatory cytokines (TNF-α, IL-1β, IL-6) and COX-2 [30]. WB of colonic tissues revealed that MPTP induction upregulated all four markers, an effect that was reversed by oral SH treatment (Figure 7A,B) (COX-2, one-way ANOVA, F (3, 8) = 67.39, *p* < 0.0001; IL-1β, one-way ANOVA, F (3, 8) = 45.72, *p* < 0.0001; TNF-α, one-way ANOVA, F (3, 8) = 6.939, *p* = 0.0129; IL-6, one-way ANOVA, F (3, 8) = 7.456, *p* = 0.0105). Analogously, the results from the midbrain showed a significant increase in pro-inflammatory proteins in the MPTP group compared to the control group. Oral SH administration significantly inhibited the elevation of pro-inflammatory proteins (Figure 7C,D) (COX-2, one-way ANOVA, F (3, 8) = 3.719, *p* = 0.0610; IL-1β, one-way ANOVA, F (3, 8) = 33.66, *p* < 0.0001; TNF-α, one-way ANOVA, F (3, 8) = 14.80, *p* = 0.0013; IL-6, one-way ANOVA, F (3, 8) = 17.85, *p* = 0.0007).

In summary, these data comprehensively clarified that SH administration effectively suppresses the production of pro-inflammatory proteins that are upregulated by the TLR4 pathway activation, thereby exerting a localized inhibitory effect on inflammation in the SN and colon.

## 3. Discussion

The gut microbiota has emerged as a critical determinant in the pathogenesis and progression of PD, with established involvement in endotoxin translocation, sustained inflammatory activation, and dysregulation of microbial metabolites [31,32,33,34]. Consequently, therapeutic strategies for PD are expanding beyond conventional dopaminergic replacement toward mechanism-based interventions that target the MGBA [3,35,36]. Aligned with this paradigm, the present study investigated the neuroprotective potential of oral SH and demonstrated that it attenuates PD-like pathology in MPTP-induced mice through coordinated modulation of the gut microbiota, intestinal barrier integrity, and the systemic LPS-TLR4/NF-κB inflammatory cascade.

The MPTP-induced subacute PD mouse model was employed in this study, as it reliably recapitulates the progressive neurodegenerative features of human PD [37,38,39]. Following peripheral administration, MPTP crosses the BBB and is metabolized by glial monoamine oxidase B to the toxic metabolite MPP^+^, which selectively induces dopaminergic neuron loss [40]. Compared with acute or chronic regimens, the subacute protocol better mirrors the gradual pathological progression of PD and was therefore selected for our experiments [41,42]. In our hands, MPTP-treated mice exhibited typical motor deficits, with reduced performance in the Rota-Rod test, pole test, and hindlimb grip strength test, and they also showed pronounced gastrointestinal dysfunction, including decreased fecal pellet output and shortened colon length. Histological examination of colon tissues revealed immune cell infiltration and epithelial damage, while brain tissue analysis confirmed the loss of TH-positive dopaminergic neurons in the striatum and cytoplasmic α-syn accumulation in the SN. These observations collectively confirm that the MPTP model effectively recapitulates the concurrent motor and gastrointestinal pathologies typical of PD. Notably, oral SH treatment significantly ameliorated both the behavioral and pathological alterations, indicating a robust protective effect in this model.

To elucidate the mechanistic basis of this protection, we first examined the impact of SH on gut microbial community structure. 16S rDNA sequencing revealed significant gut microbiota dysbiosis in MPTP-induced PD model mice. The decrease in the Shannon index suggested a trend toward microbial simplification and reduced functional redundancy, implicating impaired resilience of the microecological system to external disturbances, which is consistent with the characteristic features of gut homeostasis disruption reported in neurodegenerative diseases [43,44]. β-diversity analysis revealed a marked separation in the overall microbial composition between the model group and the control group, indicating that MPTP intervention induced systematic remodeling of the gut microbiota at the community-structure level. Following SH intervention, both microbial diversity and community structural stability were effectively restored, with the overall microbial profile converging toward that of the normal control, suggesting that SH can systematically reverse PD-associated gut microecological disturbances. Building on this foundation, we further analyzed the differential bacterial genera and SCFAs levels to investigate the potential functional mechanisms by which SH regulates the gut microbiota.

In the analysis of key differential microbiota at the genus level, we observed a significant increase in the abundance of *Lactobacillus* in the intestines of MPTP-induced PD model mice. The *Lactobacillaceae* family is widely recognized as abundant in the gut of PD patients and plays a crucial role in gut–brain interactions [45]. It is generally considered a beneficial bacterium that has been demonstrated to ameliorate both motor and non-motor symptoms [46]. However, some studies suggest that these intestinal bacteria are associated with more severe motor impairments and reduced early efficacy of the PD drug levodopa, which may effectively explain the observed phenomena [47,48]. Additionally, we noted a concurrent rise in *HT002* abundance in the PD model group. The literature confirms that *HT002* belongs to a specific genus within the *Lactobacillaceae* family, and its enrichment has been positively correlated with improved motor function in α-syn-overexpressing mouse models carrying the protective SLC39A8 variant in PD [5]. This finding suggests that *HT002* may exhibit protective potential under specific genetic or pathological conditions. Nevertheless, whether the increased abundance of *Lactobacillus* and *HT002* in the MPTP-induced acute PD model represents a compensatory response or rather a consequence of MPTP-induced disruption of microbial homeostasis remains unproven. MPTP administration caused widespread microbial dysbiosis, as evidenced by decreased α-diversity and pronounced β-diversity separation from controls. Under these conditions, the observed expansion of these bacterial taxa is more plausibly attributed to MPTP’s off-target effects on the gut ecosystem than interpreted as a marker of protection or pathology.

Meanwhile, the results demonstrated that the abundance of *Clostridium* and *Muribaculum* significantly decreased in the PD model but markedly increased after SH treatment. *Clostridium* is the predominant genus responsible for producing SCFAs in the gut [49], capable of fermenting dietary fiber to generate various functional SCFAs. The reduction in its abundance has been consistently confirmed by multiple clinical studies to be directly associated with intestinal motility disorders and increased intestinal mucosal permeability in PD patients. Research indicates that *Clostridium butyricum* (Cb) is a well-known probiotic colonizing the intestinal lumen with diverse protective effects; oral administration of Cb has been shown to reshape the gut microbiota and improve cognitive impairment in APP/PS1 transgenic mice, as well as enhance motor function deficits in a PD mouse model via the gut microbiota–GLP-1 pathway [50]. Additionally, *Muribaculum* has been proven to promote mild intestinal inflammation by regulating LPS biosynthesis [51], and its excessive proliferation exhibits a positive correlation with intestinal barrier dysfunction in PD model mice [52]. Furthermore, this genus has been reported to be involved in SCFAs production.

Based on the above changes in the bacterial community structure, we further measured the levels of SCFAs. The levels of SCFAs such as acetic acid, propionic acid, isobutyric acid, and isovaleric acid in the feces of PD mice were generally decreased [53]. However, SH intervention effectively reversed this metabolic abnormality and restored concentrations of all detected SCFAs. It is well-established that SCFAs exert intestinal protective effects by modulating macrophage polarization, promoting mucin secretion, and enhancing tight junction protein expression [20,54,55]. Recent studies have shown that butyrate upregulates ZO-1 and occludin expression via the AMPK/mTOR signaling pathway, while acetate may represent a potential therapeutic strategy for infectious diseases such as pneumonia [56]. In conclusion, our findings indicate that SH can reshape the structure of the gut microbiota, restore levels of beneficial metabolites like SCFAs, thereby inhibiting gut-derived inflammatory cascades and ultimately ameliorating PD-like pathological conditions. These findings provide a mechanistic basis for utilizing SH as a targeted microbiota-modifying intervention in early-stage PD management.

We next investigated whether SH preserved intestinal barrier function. In the MPTP model, colonic expression of tight junction proteins (ZO-1, claudin, occludin) was significantly downregulated, indicating increased intestinal permeability—a “leaky gut” phenotype consistently observed in PD patients and animal models [57]. SH treatment effectively restored the expression of these proteins and reversed barrier disruption. Such restoration is functionally relevant, as compromised intestinal barrier integrity facilitates the translocation of luminal endotoxins such as LPS into the systemic circulation [58]. Indeed, serum, colonic, and nigral LPS levels were markedly elevated in MPTP-treated mice, whereas SH administration significantly reduced them.

Elevated LPS acts as a potent activator of its pattern recognition receptor TLR4, triggering the downstream MyD88-dependent NF-κB signaling cascade, a pivotal conduit in MGBA communication [28]. In the colon of MPTP mice, we observed upregulated expression of TLR4/MyD88/NF-κB pathway components and concomitant increases in pro-inflammatory cytokines (TNF-α, IL-1β, IL-6) and COX-2. SH intervention effectively suppressed this pathway activation and the subsequent production of inflammatory mediators, thereby limiting the initiation of gut-derived systemic inflammation. This peripheral inflammatory burden, if sustained, can compromise the BBB [59,60]. In our study, MPTP challenge elevated LPS levels in the SN and reduced BBB tight junction protein expression, both of which were reversed by SH treatment. LPS entry into the central nervous system activates the TLR4/MyD88/NF-κB pathway [61,62], leading to microglial and astrocytic activation [63,64]. This inflammatory cascade is a defining feature of PD-associated neuroinflammation and is thought to contribute directly to dopaminergic neuron loss [65]. Immunofluorescence staining confirmed that SH inhibited glial cell activation in the SN and protected TH-positive neurons, further corroborating its neuroprotective action.

Collectively, our findings establish that oral SH exerts neuroprotection in MPTP-induced PD mice through a coordinated, MGBA-centered mechanism. Specifically, SH remodels gut microbiota composition, enriches SCFA-producing taxa, restores intestinal barrier integrity, reduces systemic and central LPS burden, and suppresses the TLR4/MyD88/NF-κB inflammatory axis. These interconnected events attenuate neuroinflammation, protect dopaminergic neurons, and improve both gastrointestinal and motor functions. This study thus positions SH as a promising, orally available intervention strategy for PD, particularly for individuals with gut dysbiosis and gastrointestinal dysfunction.

Several limitations warrant acknowledgement. First, the effects of SH should be verified in additional PD models, including genetic and α-syn overexpression systems, to confirm the generalizability of our observations. Second, whether SH acts through specific bacterial taxa or their metabolites, and which species are most critical for their efficacy, requires further investigation via fecal microbiota transplantation and gnotobiotic models. Third, the potential synergy between SH and existing PD therapeutics, such as L-DOPA, remains unexplored and merits future study. Nonetheless, the present findings provide compelling experimental evidence supporting the application of oral SH in the early intervention of PD and lay a foundation for its further preclinical and clinical development.

## 4. Material and Methods

### 4.1. Chemicals and Reagents

SH (1.25 M Da, purity 97.7%) was purchased from Shandong Focusfreda Biotech Co., Ltd. (Jining, China). MPTP and L-Dopa were purchased from Sigma (Burlington, MA, USA). More detailed information is provided below.

### 4.2. Animal and Experimental Design

Eight-week-old C57BL/6J mice (SPF grade, 18–20 g) with an equal number of males and females were purchased from Beijing Vital River Laboratory Animal Technology Co., Ltd. (Beijing, China). After a 7-day acclimation period under standard conditions (temperature 22 ± 2 °C, humidity 50–60%, 12 h light/dark cycle) with ad libitum access to food and water, the mice were used for experiments. All animal procedures were approved by the Institutional Animal Care and Use Committee of Shandong Academy of Pharmaceutical Sciences (Approval No. Care-2024018, Jinan, China) and conducted in strict accordance with the National Research Council’s Guide for the Care and Use of Laboratory Animals.

The SH used in this study was a food-grade material, complying with the standards for “new resource food” as regulated by the National Health Commission of China, which recommends a maximum daily intake of ≤200 mg/day for humans. Based on this recommendation and assuming a standard human body weight of 60 kg, the human-equivalent dose (HED) was calculated. To extrapolate this to mice, a conversion factor of 9.1 (based on body surface area) was applied, resulting in a maximum murine dose of 30 mg/kg/day. There is no report on the effect of oral SH on PD. Therefore, we first conducted an exploration of the dose–response relationship of SH to determine the dosage. Accordingly, three dose groups were designed to explore the dose–response relationship: a high dose (SH-H, 30 mg/kg/day), a medium dose (SH-M, 15 mg/kg/day, i.e., half of the high dose), and a low dose (SH-L, 7.5 mg/kg/day, i.e., one-quarter of the high dose).

To identify an effective therapeutic dose, mice were randomly divided into six groups (*n* = 6 per group): Control, MPTP (model), L-DOPA (positive control, 15 mg/kg/day), SH-L (7.5 mg/kg/day), SH-M (15 mg/kg/day), and SH-H (30 mg/kg/day). All treatments were administered orally via gavage daily. For the first four weeks, the Control and MPTP groups received saline, while the other groups received their respective treatments (L-DOPA or SH). In the fifth week, PD was induced in all groups except the Control group by intraperitoneal injection of MPTP (25 mg/kg/day) for seven consecutive days; the Control group received an equivalent volume of PBS. Body weight was recorded every five days throughout the study. In the final week, gastrointestinal function and behavioral tests were performed. All mice were euthanized at the end of the fifth week for tissue collection and subsequent analyses.

### 4.3. Behavioral Tests

Motor function was assessed via three behavioral tests (1 h apart) after a 7-day pre-training period for acclimation.

#### 4.3.1. Rota-Rod Test

The Rota-Rod test was performed 24 h post-dose. The rod accelerated from 10 to 40 r/min over 300 s (cutoff: 300 s) [66]; fall latency was recorded. Each mouse was tested three times at 1 h intervals per day, and the 3 d average was used for analysis.

#### 4.3.2. Pole Test

For the pole test, a gauze-wrapped metal rod (50 cm × 8 mm) was mounted vertically on a bedding-covered base, with a 1 cm ball at the top for forelimb grasping. Mice were placed head-up on the ball and released; descent time was recorded. Training preceded testing, and three trials were performed at 1 h intervals per mouse.

#### 4.3.3. Hindlimb Clasping Test

The hindlimb clasping test was performed on day 31 to evaluate neurodegenerative progression. Each mouse was lifted by the tail and suspended away from any surfaces, and hindlimb posture was observed for 10 s. Clasping severity was scored on a 0–3 scale based on the position of both limbs during suspension [67]: 0, both hindlimbs consistently splayed outward; 1, one limb adducted toward the abdomen for >50% of the observation period; 2, both limbs adducted for >50% of the period; 3, both limbs fully retracted and touching the abdomen.

#### 4.3.4. Fecal Pellet Output

To assess intestinal motility and fecal parameters, mice were fasted for 2 h and then transferred to clean transparent cages for a 2 h collection period. Fecal pellets were enumerated and immediately weighed to determine wet weight, followed by drying at 85 °C for 24 h to obtain dry weight; fecal water content was derived from the wet–dry differential. For dynamic motility evaluation, stool output was recorded at 5 min intervals over 20 min. Upon sacrifice, the colon was excised and its length from the cecal end to the anal verge was measured.

### 4.4. Tissue Preparation

Following behavioral tests, mice were deeply anesthetized with sodium pentobarbital (50 mg/kg, i.p.). Blood was collected from the retro-orbital venous plexus using sterile glass capillaries, allowed to clot at room temperature for 30 min, and centrifuged at 3000× *g* for 15 min at 4 °C. The separated serum was stored at −80 °C until use. Mice were then euthanized by cervical dislocation. The brain was rapidly removed, and the midbrain (including SN) was dissected on ice. The colon was excised, flushed with ice-cold PBS, and the mucosa was scraped. All tissue samples were snap-frozen in liquid nitrogen and stored at −80 °C. Fecal samples were collected prior to sacrifice and stored at −80 °C for subsequent analyses.

### 4.5. Hematoxylin and Eosin Staining

According to our previous study, colon sections embedded in paraffin were stained with hematoxylin and eosin (H&E). Histological scoring was conducted by two blinded researchers. The scoring criteria for tissue damage were as follows [68]: 0, no damage; 1, lymphoepithelial lesions; 2, focal ulceration or mucosal erosion; 3, extensive mucosal damage involving deep structures of the intestinal wall. The scoring for inflammatory cell infiltration was based on the following criteria [68]: 0, few inflammatory cells in the lamina propria; 1, increased infiltration of inflammatory cells into the lamina propria; 2, infiltration of inflammatory cell clusters into the submucosa; 3, transmural infiltration of inflammatory cells. The histological score was determined by combining the scores for tissue damage and inflammatory cell infiltration. Each section was calculated based on three randomly selected fields.

### 4.6. Immunofluorescence Staining

Mice (*n* = 3) were anesthetized and perfused with saline and 4% paraformaldehyde in phosphate buffer. Colonic and brain tissues were dissected, post-fixed overnight in 4% paraformaldehyde, and cryoprotected in 30% sucrose/PFA, then paraffin-embedded, sectioned at 5 μm, and subjected to antigen retrieval (citrate buffer, pH 6.0). Sections were blocked with 3% BSA (Servicebio, Wuhan, China) and incubated overnight at 4 °C with primary antibodies: anti-TH (1:1000), anti-Iba-1 (1:1000), and anti-GFAP (1:1000) (all Servicebio), and anti-ZO-1 (1:100; Thermo Fisher, Waltham, MA, USA). After secondary antibody incubation (FITC-goat anti-mouse, 1:400; goat anti-rabbit-CY3, 1:300; goat anti-mouse-CY3, 1:300; Servicebio), nuclei were counterstained with DAPI. Images were captured under a fluorescence microscope (Nikon Eclipse C1, Tokyo, Japan) and analyzed with Image Pro Plus 6.0.

### 4.7. Enzyme-Linked Immunosorbent Assay (ELISA)

The concentrations of TNF-α, IL-6, and LPS in mouse serum were determined using commercial ELISA kits (Shanghai Jianglai Industrial Co., Ltd., Shanghai, China), with catalog numbers TNF-α (Cat# JL10208-96T), IL-6 (Cat# JL14113-96T), and LPS (Cat# JL12996-96T). All assays were carried out according to the manufacturer’s instructions, and the concentrations of the target analytes were calculated by interpolation from the corresponding standard curves.

### 4.8. Fecal DNA Extraction and 16S rDNA Sequencing

For microbiota sequencing analysis, mice from each group were randomly selected at week 4. Fresh fecal pellets from each mouse were collected, immediately placed into sterile EP tubes, and immediately frozen at −80 °C for further analysis. Microbial genomic DNA was extracted from the fecal samples using the E.Z.N.A.^®^ Stool DNA Kit (D4015, Omega Inc., Norcross, GA, USA) according to the manufacturer’s instructions. The V3–V4 region of the 16S rDNA was amplified using paired primers (341F (5′-CCTACGGGNGGCWGCAG-3′) and 805R (5′-GACTACHVGGGTATCTAATCC-3′)) [69]. Subsequently, the amplified 16S rDNA fragments were sequenced using the Illumina NovaSeq platform (San Diego, CA, USA) according to the instructions provided by LC-Bio. Paired-end reads were merged using FLASH, version 1.2.11. Noise reduction (generating ASV tables), feature data summary, taxonomic analysis (mapping reads to Greengenes 13_8), and generation of taxonomy tables were performed using dada2 within QIIME2 (2022-2 version) [70]. The taxonomy tables were purified by eliminating low-abundance taxa (average relative abundance <0.1% in each group; prevalence rate of 10%). Filtered taxonomy tables were used for microbiota analysis. α diversity, β diversity, PCoA, permutational multivariate analysis of variance (PERMANOVA), and triplot analysis were conducted using the microbiota analysis software package EasyMicroPlot (0.5.1 version) [71].

### 4.9. Western Blotting

For total protein extraction, tissues were lysed using RIPA lysis buffer (P0013B, Beyotime, Shanghai, China) mixed with a protease and phosphatase inhibitor cocktail (P1045, Beyotime, Shanghai, China). The lysates were centrifuged at 12,000× *g* for 20 min at 4 °C to obtain total protein. Protein concentrations were determined using a BCA protein assay kit (P0010, Beyotime, Shanghai, China). WB was performed using SDS-PAGE. Membranes were incubated at 4 °C overnight with the following primary antibodies: rabbit anti-β-actin antibody (1:50,000, AC026, Abclonal, Wuhan, China), mouse anti-TH antibody (1:1000, A0028, Abclonal, Wuhan, China), rabbit anti-TNF-α antibody (1:1000, A20851, Abclonal, Wuhan, China), rabbit anti-IL-1β antibody (1:1000, A11369, Abclonal, Wuhan, China), rabbit anti-IL-6 antibody (1:1000, A0286, Abclonal, Wuhan, China), goat anti-COX-2 antibody (1:1000, A1253, Abclonal, Wuhan, China), mouse anti-TLR4 antibody (1:1000, A5258, Abclonal, Wuhan, China), rabbit anti-MyD88 antibody (1:400, A0980, Abclonal, Wuhan, China), rabbit anti-IκB-α antibody (1:1000, A24742, Abclonal, Wuhan, China), rabbit anti-ZO-1 antibody (1:1000, A0659, Abclonal, Wuhan, China), mouse anti-claudin-1 antibody (1:1000, Abclonal, Wuhan, China), rabbit anti-occludin antibody (1:1000, Abclonal, China), and mouse anti-claudin-5 antibody (1:1000, A10207, Abclonal, Wuhan, China). The membranes were then incubated with appropriate secondary antibodies, including HRP-conjugated anti-mouse antibody (1:2000, AS003; clone), HRP-conjugated anti-rabbit antibody (1:2000, AS014; clone), and HRP-conjugated anti-goat antibody (1:2500, AS031; Abclonal) at room temperature for 2 h. WB were visualized using a LAS4000 chemiluminescence system (Fujifilm, Tokyo, Japan) and analyzed for density using ImageJ software, version 1.54i.

### 4.10. Statistical Analysis

Statistical analysis was performed using GraphPad Prism 8.0.2 (GraphPad Software, San Diego, CA, USA, www.graphpad.com). Data were expressed as the mean ± standard deviation (SD). Multiple group comparisons were analyzed using one-way analysis of variance (ANOVA) followed by Tukey’s post hoc test. *p*-values less than 0.05 were considered statistically significant.

## Data Availability

The original contributions presented in this study are included in the article/Supplementary Materials. Further inquiries can be directed to the corresponding authors.

## Supplementary Materials

The following supporting information can be downloaded at: https://www.mdpi.com/article/10.3390/ijms27156573/s1.

## Abbreviations

The following abbreviations are used in this manuscript:

BBB	blood–brain barrier
CCK-8	cell counting kit-8
CNS	central nervous system
COX-2	Cyclooxygenase-2
ECM	extracellular matrix
ELISA	enzyme-linked immunosorbent assay
GAG	glycosaminoglycan
GSH	reduced glutathione
GSSG	oxidized glutathione
H&E	hematoxylin and eosin
HA	hyaluronic acid
HABP	hyaluronic acid binding protein
IF	immunofluorescence
IHC	immunohistochemistry
IL-1β	interleukin-1 beta
IL-6	interleukin-6
LBs	Lewy bodies
LPS	lipopolysaccharide
MPTP	1-methyl-4-phenyl-1,2,3,6-tetrahydropyridine
PBS	phosphate-buffered saline
PD	Parkinson’s disease
SCFAs	short-chain fatty acids
SH	sodium hyaluronate
SN	substantia nigra
SNpc	substantia nigra pars compacta
TH	tyrosine hydroxylase
TNF-α	tumor necrosis factor alpha
WB	Western blot
α-syn	α-synuclein
