# Supplementary material for: Oral Sodium Hyaluronate Reshapes Gut Microbiota Composition and Suppresses the LPS-TLR4/NF-κB Pathway to Exert Neuroprotection in MPTP-Induced Parkinson’s Disease
Source: Int J Mol Sci. 2026 Jul 23;27(15):6573. doi: 10.3390/ijms27156573 (PMC13466556; doi:10.3390/ijms27156573)
Supplement: Supplementary file 1 [file ijms-27-06573-s001.zip › ijms-4399318-supplementary.pdf]

Supplementary information

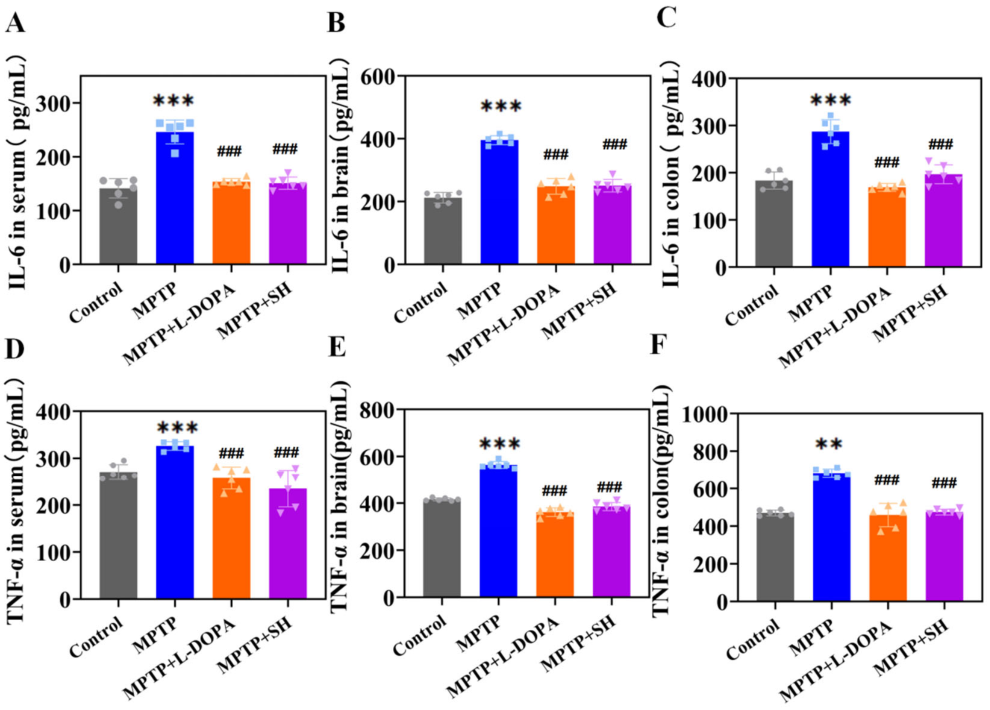

Figure S1 Proinflammatory cytokine content detection. (A-C) Levels of TNF-α in serum, the midbrain containing the SN and the colon. (**D-F**) Levels of IL-6 in serum, the midbrain containing the SN and the colon. n = 6 for each group. Data are presented as mean ± SD. *** $p < 0.001$, versus the control group; $^{###}$ $p < 0.001$ versus the MPTP group.

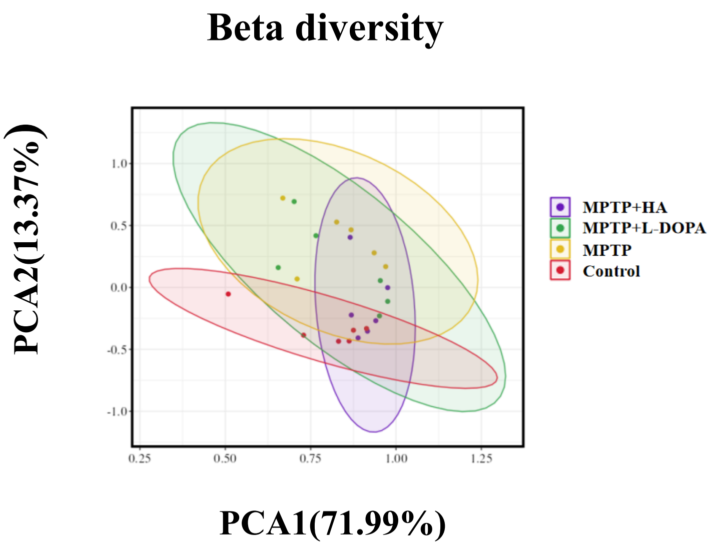

Figure S2. Principal Component Analysis (PCA) of gut microbial communities based on weighted UniFrac distances.

Table S1

Relative abundance of microbiota at different taxon levels

Taxonomic level	Relative abundance				Pvalue		
	Control	MPTP	MPTP+L-DOPA	MPTP+SH	Control vs MPTP	MPTP+L-DOPA vs MPTP	MPTP+SH vs MPTP
**Phylum**							
Firmicutes	52.2545	77.6136	64.5515	53.1271	0.0192*	0.1911	0.0341*
Bacteroidota	35.8489	10.1971	25.2604	36.7598	0.0045**	0.0184*	0.0108*
Patescibacteria	0.9873	1.7778	1.0646	1.5793	0.0640	0.0483*	0.6396
Cyanobacteria	0.2999	0.0593	0.1567	0.3191	0.1531	0.2499	0.0171*
**Family**							
Lactobacillaceae	30.3706	70.5059	41.4723	26.8258	0.0045**	0.2326	0.0015**
Muribaculaceae	29.2892	8.9861	21.0264	30.9740	0.0073**	0.1941	0.0178*
Lachnospiraceae	7.4201	1.6483	7.9201	5.1452	0.0499*	0.0825	0.0646
Clostridia_UCG-014_unclassified	1.4851	2.0572	4.9619	13.1174	0.2872	0.4456	0.0187*
Clostridiales_unclassified	1.9802	0.1994	3.2633	1.7338	0.2769	0.1312	0.0206*
Rikenellaceae	0.9825	0.6204	1.5517	2.2541	0.4854	0.3113	0.0036**
Ruminococcaceae	1.2261	0.2367	1.8220	1.2139	0.2771	0.1408	0.0188*
Prevotellaceae	1.8000	0.1681	0.7657	0.6779	0.0289*	0.2693	0.0138*
Bacteroidaceae	2.4269	0.1159	0.2720	0.4244	0.1533	0.0415*	0.0128*
**Genus**							
Muribaculaceae_unclassified	26.4885	7.4494	18.1392	27.4127	0.0125*	0.1923	0.0090**
Others	10.3359	4.2790	8.2065	6.8536	0.0005***	0.0626	0.0068**
Akkermansia	3.7571	3.6720	0.5686	0.3314	0.2872	0.2952	0.0187*
Lachnospiraceae_unclassified	2.5475	0.1435	2.1032	0.6769	0.0197*	0.0597	0.4380
Muribaculum	2.4462	0.9249	1.9162	2.5038	0.0302*	0.0754	0.0090**
Bacteroides	2.4269	0.1159	0.2720	0.4220	0.0304*	0.8812	0.5640
Lachnospiraceae_NK4A136_group	2.3000	0.4375	2.5946	3.2042	0.2769	0.0645	0.0206*
Clostridiales_unclassified	1.9802	0.1994	3.2633	1.7338	0.0640	0.0482*	0.6396
Lactobacillus	1.9467	36.5976	16.8236	10.4835	0.0014**	0.0601	0.0076**
Enterorhabdus	1.1103	1.4409	2.2060	2.5284	0.0494*	0.0692	0.0586
Candidatus_Saccharimonas	0.9873	1.7778	1.0641	1.5793	0.1533	0.0130*	0.0140*
Eubacterium]_coprostanoligenes_group_unclassified	0.7283	0.0898	0.3118	0.2884	0.1034	0.0233*	0.0013*
Alloprevotella	0.3851	0.1061	0.6399	0.5517	0.3621	0.0429*	0.0060**
Escherichia-Shigella	0.3025	0.2229	0.4184	0.1458	0.0479	0.1487	0.0138*
HT002	0.2904	5.1400	1.1475	1.3460	<0.0001****	<0.0001****	0.0001***
Bacteroidota_unclassified	0.2599	0.0585	0.9047	1.6057	0.1059	0.0636	0.0431*

* $p < 0.05$, ** $p < 0.01$, *** $p < 0.001$. Statistical analysis by the Kruskal-Wallis multiple comparison test with the post-hoc Mann-Whitney U test.
